# Qualitative and quantitative characteristics of CEUS for renal cell carcinoma and angiomyolipoma: a narrative review

**DOI:** 10.1007/s40477-023-00852-x

**Published:** 2024-01-19

**Authors:** Piervito Dipinto, Vittorio Canale, Rocco Minelli, Marco Alex Capuano, Orlando Catalano, Giovanni Battista Di Pierro, Umberto Anceschi, Sisto Perdonà, Antonio Tufano

**Affiliations:** 1 Department of Maternal-Child and Urological Sciences, Policlinico Umberto I Hospital, Sapienza University of Rome, 00162 Rome, Italy; 2 Department of Medicine and Health Sciences “Vincenzo Tiberio”, University of Molise, Via Francesco De Sanctis, 1, 86100 Campobasso, CB Italy; 3 Radiology Unit, Varelli Diagnostic Institute, 80126 Naples, Italy; 4 Department of Urology, IRCCS “Regina Elena” National Cancer Institute, 00144 Rome, Italy; 5 Fondazione “G. Pascale” IRCCS, 80131 Naples, Italy

**Keywords:** Contrast-enhanced ultrasound (CEUS), Renal cell carcinoma (RCC), Angiomyolipoma (AML), Clear cell RCC (ccRCC)

## Abstract

Incidental findings of renal masses are increasing. However, a substantial portion of surgically treated renal masses turn out to be benign on histopathological examination. Thus, there is a clear need for improved pre-surgical assessment to minimize unnecessary invasive procedures. The challenge intensifies when distinguishing between renal cell carcinoma (RCC) and angiomyolipoma (AML) in renal lesions smaller than 4 cm with minimal adipose tissue. In such cases, contrast-enhanced ultrasound (CEUS) has emerged as a valuable diagnostic tool, by utilizing both qualitative and quantitative parameters. Quantitative measures offer objectivity, reliability, and reproducibility compared to qualitative parameters, enabling the characterization of RCC subtypes and differentiation from AML. Qualitative features as enhancement pattern, degree, and peak were less helpful in distinguishing triphasic minimal fat AML (TAML) from epithelioid AML (EAML), with the pseudocapsule sign potentially being the only distinguishing qualitative feature. The pseudocapsule sign was more frequently observed in ccRCCs (38.0%) than in AMLs (15.6%). Moreover, it was detected in 40.0% of EAMLs and 34.5% of ccRCCs but not in TAMLs due to similar growth patterns between EAMLs and low-grade ccRCCs. Quantitative measures such as the time-to-peak (TTP) ratio can further enhance diagnostic accuracy and also TOC ratio should be considered, as it was higher in clear cell RCCs (ccRCCs) and in EAMLs compared to TAMLs, indicating behavior similar to ccRCCs. However, CEUS remains an operator-dependent exam.

## Introduction

The use of abdominal cross-sectional imaging has grown in the last decades, leading to a consistent increase in the diagnosis of incidental renal masses. However, approximately 10–17% of surgically removed renal masses turn out to be benign [1]. Of those, between 2 to 6% are identified as angiomyolipoma’s (AML) upon final histopathological examination [2, 3]. Therefore, a correct preoperative characterization of renal masses is crucial to avoid unnecessary treatments. AML are hamartomatous benign tumors composed of newly formed vessels, smooth muscle cells, and well-differentiated adipose tissue. On renal ultrasound (US), they typically present as a hyperechoic mass, while on CT scans, they usually exhibit macroscopic fat (less than −20 HU) [4, 5]. However, imaging features may overlap between renal cell carcinoma (RCC) and AML, especially when they are fat-poor or are smaller than 4 cm (cT1a) [6]. In these scenario, differential diagnosis might be challenging, and contrast-enhanced ultrasound (CEUS) has emerged as a valid diagnostic tool, offering several advantages such as the absence of radiation exposure, nephrotoxicity, or electromagnetic interference of metal implants. However, qualitative analysis and interpretation of CEUS are operator-dependent and exhibit limited reproducibility. Qualitative features such as heterogeneous enhancement, rapid wash-out and the presence of a pseudo capsule sign, are often observed in RCCs [7–10]. Conversely, AMLs typically display slow centripetal enhancement, homogeneous peak enhancement, and gradual wash-out [11]. Basing on the proportion of fat, AML are typically categorized into two subtypes, rich-fat AML and minimal fat AML subtypes. Notably, the latter is divided into epithelioid AML (EAML) and triphasic minimal fat AML (TAML). EAML is characterized by atypical epithelioid cells and minimal fat, requiring surgical treatment, due to its malignant potential. On the other hand, TAML typically does not require surgery unless it becomes symptomatic [12–14]. Therefore, distinguishing TAML and EAML is extremely important in clinical decision making. Aim of the current review is to describe the qualitative and quantitative characteristics of AML and RCC on CEUS.

## Qualitative evaluation of RCC and AML on CEUS

Qualitative parameters commonly employed to differentiate between RCC and AML are degree of enhancement and enhancement patterns. The degree of enhanchement assesses how much the mass enhances compared to the adjacent cortex. It can be categorized as hyperenhancement (greater than cortex), isoenhancement (equal to the cortex), hypoenhancement (lesser than cortex), or non-enhancement. Instead, enhancement pattern considers dynamic changes in enhancement during wash-in, wash-out phases and the presence of a pseudocapsule sign. The enhancement patterns displayed by renal tumors can be either heterogeneous or homogeneous, depending on how contrast diffuses within the tumors in relation to their vascularization profile. The pseudocapsule sign, characterized as an accentuated border of peritumoral tissue, becomes more distinct in the parenchymal (or late) phase [15]. In a study by Chen et al., involving the analysis of over 100 renal masses, RCCs were found to exhibit a typical hyperenhancement (79%), homogeneous enhancement (66.7%), early elimination of contrast compared to the peripheral cortex during the late phase (77.8%), and peripheral rim-like enhancement (55.6%). In contrast, AMLs often displayed isoenhancement (61.9%), homogeneous enhancement (85.7%), and slower contrast disappearance during the late phase [16]. XuZF et al. also considered the timing of tumor enhancement, showing no significant difference between RCC and AML concerning renal cortex enhancement [17]. In this case, RCCs demonstrated heterogeneous enhancement (74.2%), whereas AMLs exhibited more homogeneous enhancement (87.9%). Rims of peritumoral tissue enhancement were observed in 79.6% of RCCs and 3.0% of AMLs. Cao et al. emphasized the significance of enhancement perilesional edge-like enhancement and rapid wash-out as key features of RCCs, with the rate of perilesional edge enhancement being approximately 76.7% in RCCs, similar to rates in other studies such as Xu et al. and Van Oostenbrugge et al. [18–20]. According to the authors, the CEUS characteristics of RCCs remain a subject of debate, influenced by factors like tumor size. Heterogeneous enhancement in RCCs was primarily observed in tumors larger than 4 cm, while a more homogeneous pattern could be observed in small RCCs, which grow slowly and rarely exhibit internal necrosis [21]. Several studies have associated specific characteristics with RCCs, including rapid wash-out and the presence of the perilesional edge [16, 17]. These features are attributed to tumor growth, causing compression, ischemia, and subsequent necrosis of adjacent parenchyma, followed by the deposition of fibrous tissue. Additionally, heterogeneous enhancement and early-stage hyperenhancement on CEUS are often linked to the likelihood of rapidly growing RCCs, characterized by thin-walled immature blood vessels and many arterial-venous fistulas [22, 23]. On the other hand, AMLs often demonstrated iso-enhancement on CEUS, with slow contrast accumulation and gradual wash-out [16, 17]. This is likely due to blood vessel abnormalities, including thickened vessel walls and variable amounts of slowly growing adipose tissue, with less frequent necrosis and ischemia in the adjacent renal parenchyma [24]. These characteristics explain a more frequent homogeneous pattern and the rare presence of the perilesional border, unlike RCCs [25]. Occasionally, AMLs, especially those larger than 4 cm, may display spontaneous hemorrhages with a heterogeneous pattern on CEUS [18]. One limitation of these studies is that statistical analysis was often performed without differentiating the different subtypes of RCC. The different subtypes of RCC can significantly modify the characteristics of CEUS, with the degree of enhancement of ccRCC being higher than that of papillary RCC (pRCC) and chromophobe RCC (chRCC) due to the rich blood supply linked to ccRCC, justifying ccRCC as the most aggressive variant of renal cell cancer [26, 27].

## Quantitative evaluation of RCC and AML on CEUS

The development of new softwares and quantitative parameters in contrast-enhanced ultrasound (CEUS) has shown promising results to improve the accuracy of differentiating angiomyolipoma (AML) from various subtypes of renal cell carcinoma (RCC). Li CX et al. introduced a parameter known as ROImax, which represents the region of interest with the maximum intensity within the tumor [28]. This parameter includes time-related measurements, such as rise time (RTmax, the time taken for enhancement intensity to go from 10 to 100% of the maximum value), time to peak (TTPmax, the time from the first appearance of the contrast agent in any ROI to the peak point of the specific ROI), and mean transit time (mTTmax, the wash-out rate of the contrast agent in the ROI). Notably, these time-related parameters were consistently shorter in all RCC subtypes when measured using ROImax compared to the corresponding measurements obtained using ROITumor, which represents the region of interest encompassing the entire tumor. However, there were no significant differences in these parameters between ROImax and ROITumor measurements in AML cases. These findings are attributed to the high rate of necrosis and the heterogeneous distribution of vessels in RCCs. The study also examined other quantitative parameters such as maximum intensity (IMAX, representing the degree of enhancement of the ROI relative to the renal parenchyma at the time of the peak) and area under the curve (AUC, representing the total amount of blood perfusion of the ROI during the analysis period) to conduct statistical analyses between RCC subtypes and AML. The authors found that ΔIMAX and ΔAUC in ccRCC were significantly higher than those observed in chRCC and pRCC subtypes, which is attributed to the vascularization of ccRCC with fistulas, resulting in rapid wash-in and wash-out. Furthermore, ΔIMAX and ΔAUC of ccRCC were also higher than those obtained in AML cases. In contrast, ΔIMAX and ΔAUC of AML were higher than those of chRCC and pRCC. For this reason, the authors underline the importance of using ΔmTT to distinguish RCC, independently of subtypes, from AML, because ΔmTT of AML was significantly higher than that of RCC. Additionally, ΔIMAX and ΔAUC can be used to differentiate between subtypes of RCC. Similar results were obtained in a study conducted by Liu H et al., in which various indices, including peak intensity (ΔPI), slope (ΔSL), area under the wash-out curve (ΔAUC), area under the washin curve (ΔAWI), area under the wash-out curve (ΔAWO), time to reach peak intensity (ΔTTP), and ΔmTT, showed significant differences between RCC and AML. Increased ΔPI, ΔSL, ΔAUC, and ΔAWO could effectively differentiate ccRCC from both pRCC and chRCC, demonstrating reliable diagnostic efficiency [29].

## “Minimal fat” hypoechoic AML: a diagnostic dilemma

Among minimal fat AMLs, there are a few that can appear hypoechoics. These are predominantly composed of smooth muscle and can mimic ccRCCs on imaging. Lu et al. conducted a study to differentiate between these "minimal fat AMLs" and ccRCC. AMLs were found to commonly exhibit a centripetal enhancement pattern, while ccRCCs typically displayed full enhancement. At peak enhancement, all AMLs showed homogeneous enhancement, while only 27.5% of ccRCCs did. The pseudocapsule sign was more frequently observed in ccRCCs (38.0%) than in AMLs (15.6%) [30]. A quantitative analysis considered three parameters: Imax, RT and TTP. Imax was normalized using the tumor-to-cortex enhancement ratio (TOC ratio) due to depth-related variations. RT and TTP showed no significant differences between AML and ccRCC, while the TOC ratio was higher in ccRCCs with minimal fat due to differences in vessel aspect ratios and vascularization [30]. According to Xu ZF et al., considering all variants of AML, no significant difference in the degree of enhancement was found between AML and ccRCC, unlike AML and ccRCC with minimal fat [17]. Therefore, from a qualitative perspective, centripetal and homogeneous enhancement were the main characteristics of "minimal-fat AML" on contrast-enhanced ultrasound (CEUS). From a quantitative perspective, a lower degree of enhancement distinguished hypoechoic AMLs from ccRCCs. Lu et al. conducted a comprehensive investigation of minimal fat renal AML, distinguishing between epithelioid AML (EAML) and triphasic minimal fat AML (TAML) [31]. EAML, primarily composed of epithelioid cells with less than 10% fat, had a higher malignant potential, often requiring surgery as RCC. In contrast, TAML, characterized by thick-walled blood vessels, smooth muscle, and limited adipose tissue, typically did not necessitate surgical intervention unless symptomatic [31, 32]. Qualitative features like enhancement pattern, degree, and peak were less helpful in distinguishing TAML from EAML, with the pseudocapsule sign potentially being the only distinguishing qualitative feature. It was detected in 40.0% of EAMLs and 34.5% of ccRCCs but not in TAMLs due to similar growth patterns between EAMLs and low-grade ccRCCs. Indeed, histologically EAML can closely resemble low-grade ccRCC [31, 33]. No significant differences were found between EAML and TAML regarding time-related quantitative parameters like RT and TTP, which were significantly shorter in ccRCCs. However, the TOC ratio should be considered, as it was higher in ccRCCs and EAMLs compared to TAMLs, indicating behavior similar to ccRCCs [31]. AUC value may also be a useful parameter when used to evaluate specific predictive models [34]. The qualitative and quantitative characteristics analyzed by CEUS among the main studies are summarized in Table 1.

**Table 1:** Summary of CEUS characteristics among major eligible studies in differentiating AML versus RCC

AML vs RCC	Year	Single center/multi center	Design of the study	Number of patients	Number of RCC and AML	Size of renal lesions (DT)	Qualitative characteristics of RCCs	Qualitative characteristics of AML	Quantitative analysis of RCC and AML		Ultrasound equipment
Xuet al. [17]	2010	Single center	Retrospective	119	90 RCC 29 RAML	RCC 4.2 cm; AML 3.7 cm	Heterogeneous improvement (74.2%); hyperenhancement (88.2%) and rapid flushing (71.0%); perilesional edge (79.6%)	Homogeneous improvement (87.9%); sustained hyperpotentiation or isopotentiation (78.8%); perilesional edge (3.0%)	No	No	Acuson Sequoia 512 scanner and Aplio XV machine; Contrast SonoVue
Cao et al. [18]	2020	Single center	Retrospective	165	150 RCCs 22 anti-money laundering	RCC 4.04 ± 2.21 cm; AML 3.47 ± 2.22 cm	Heterogeneous improvement (52.0%); quick wash (48.7%), hyperenhancement (74.7%), and quick wash (56.0%); perlesional edge (76.7%)	Homogeneous improvement (77.3%); synchronous wash-in (50.0%), isoenhancement (45.5%) or hypoenhancement (36.4%); slow washing (86.4%); perlesional edge (9.1%)	No	No	Aplio500; contrast SonoVue
Chen et al. [16]	2015	Single center	Retrospective	99	79 RCC 20 anti-money laundering	< 3 cm	Homogeneous improvement (66.7%); hyperenhancement (79.0%), rapid flushing (45.7%), and rapid flushing (77.8%); perlesional edge (55.6%)	Homogeneous improvement (85.7%); synchronous wash-in (52.4%), isoenhancement (61.9%), slow wash-out (47.9%); perilesional edge (9.5%)	No	No	Acuson S2000; contrast SonoVue
Cao et al. [27]	2022	Single center	Retrospective	151	110 ccRCC, 12 pRCC, 9 chRCC 20 anti-money laundering	< 4 cm	Homogeneous improvement (67.2%); hyperenhancement (68.7%), synchronous/slow flushing (51.1%), and fast flushing (64.9%); perilesional edge (80.9%); EI of ccRCC > pRCC and chRCC	Homogeneous improvement (95.0%); wash in synchronous/slow (90.4%), iso/hypoenhancement (90.0%), wash-out synchronous/slow (95.0%); perilesional edge (5.0%)	No	No	Aplio500; contrast SonoVue
Li et al. [28]	2016	Single center	Retrospective	411	280 ccRCC, 28 pRCC, 33 chRCC 88 anti-money laundering	RCC 3.7 ± 1.7 cm; anti-money laundering 3.9 ± 2.1 cm	NO	NO	ΔmTT of LMA > pRCC = ccRCC = chRCC; ΔIMAX and ΔAUC of ccRCC > AML > pRCC and chRCC		system; contrast SonoVue
Liu et al. [29]	2022	Single center	Retrospective	97	71 ccRCC, 7 pRCC, 7 chRCC 12 anti-money laundering	< 4 cm	NO	NO	ΔPI, ΔSL, ΔAUC, ΔAWI, ΔAWO, ΔTTP and ΔmTTs differ significantly between RCC and AML; ΔPI, ΔSL, ΔAUC, and ΔAWO of ccRCC > pRCC and chRCC		Aplio500; contrast SonoVue

AML with minimal fat vs ccRCC	Year	Single center/multi center	Design of the study	Number of patients	Number of RCC and AML	Size of the renal lesion (DT)	Qualitative characteristics of ccRCC	Qualitative characteristics of AMLs with minimal fat	Quantitative analysis of ccRCCs and AML with minimal fat	Ultrasound equipment
Lu et al. [30]	2015	Single center	Retrospective	197	149 ccRCC 35 anti-money laundering	ccRCC 3.5 ± 1.9 cm; anti-money laundering 3.9 ± 2.0 cm	Entire improvement (76.8%); heterogeneous enhancement (72.5%); perilesional edge (38.0%)	Centripetal enhancement (71.9%); homogeneous valorisation (100%); perilesional edge (15.6%)	RT and TTP no significant difference; Higher TOC ratio in ccRCCs compared to AMLs	system; contrast SonoVue

EAML vs. TAML vs. RCC	Year	Single center/multi center	Design of the study	Number of patients	Number of RCC and AML	Size of the renal lesion (DT)	Qualitative characteristics of RCCs	Qualitative characteristics of TAMLs	Characteristic qualitative of the EAML	Quantitative analysis of RCCs and AML with minimal fat	Ultrasound equipment
Lu et al. [31]	2015	Single center	Retrospective	153	133 RCC; 15 EAML; 25 TAML	RCC 3.2 ± 1.4 cm; EAML 3.4 ± 1.8 cm; TAML 3.6 ± 1.5 cm	Entire improvement (81.4%); heterogeneous enhancement (56.6%); perilesional edge (34.5%)	Centripetal enhancement (84.0%); homogeneous valorisation (100%); perilesional edge (0.0%)	Centripetal enhancement (73.3%); homogeneous valorisation (100%); perilesional edge (40.0%)	Shorter RT and TTP in RCCs, but no difference between EAML and TAML; Higher TOC ratio in RCCs and EAMLs compared to TAMLs	System E 9; contrast SonoVue

## Conclusions

CEUS presents itself as a valuable tool for distinguishing between RCCs and AMLs, particularly the challenging AMLs with minimal fat. It achieves this by analyzing various factors including enhancement patterns, degree of enhancement, the presence or absence of the pseudocapsule sign, and wash-in and wash-out times. Additionally, quantitative measures like the TOC ratio can provide useful diagnostic information. It's worth noting that while CEUS offers valuable insights, CT and MRI remain the primary imaging modalities for diagnosing malignant or benign renal lesions. CEUS does have limitations, notably its reliance on operator expertise. However, ongoing technological advancements and growing proficiency in its use continue to enhance the utility and reliability of CEUS in clinical practice.

## Data Availability

Not available.
